# First Day

**Published:** 1881-06

**Authors:** 


SUPPLEMENT.

THE

CHICAGO MEDICAL

JOURNAL & EXAMINER.

Vol. XLII.—JUNE, 1881.—No. 6.

THE

### AMERICAN MEDICAL ASSOCIATION

### PROCEEDINGS OF THE THIRTY-SECOND ANNUAL MEETING,

AT RICHMOND, VIRGINIA.

### FIRST DAY—Tuesday, May 3, 1881.

The American Medical Association met in its Thirty-second Annual Session at 11 o'clock A. M., at Mozart Hall, Tuesday, May 3, 1881.

The Association was called to order by Dr. Frank Cunningham, Chairman of the Local Committee of Arrangements.

Dr. H. D. Holton, of Vermont, Vice President of the Association, occupied the chair, with Dr. William B. Atkinson, of Philadelphia, Pennsylvania, acting as Secretary.

The exercises opened with prayer by the Right Rev. J. J. Keane.

Dr. Cunningham then in appropriate language introduced Governor F. W. M. Holliday, who had consented to deliver the address of welcome.

The address of Governor Holliday was, like all of his efforts, worthy both of the man and the occasion. In fitting terms he alluded to the importance of the great gathering, and complimented the physicians of the country upon the high tone and usefulness of the profession, and bade every member a warm and heartfelt welcome to the capital city of the Old Dominion.

The Secretary next called the roll of members who had registered.

A motion to dispense with the calling of the roll was laid on the table.

The names read were, on motion of Dr. Toner, all confirmed in their membership.

On motion of Dr. Brodie, the ex-Presidents were escorted to seats on the platform.

Dr. Cunningham, from the Committee of Arrangements, read to the Association invitations from the Commercial, Richmond, and Westmoreland Clubs tendering the hospitalities of these Clubs to the members during the session of the Association. The invitations were accepted, and, on motion of Dr. D. J. Roberts, the thanks of the Association tendered for the same.

After hearing the report of the Committee of Arrangements, the Secretary read letters of regret for non-attendance from ex-Presidents J. Marion Sims, of New York, and W. O. Baldwin, of Alabama; Vice-Presidents W. H. Anderson, of Alabama, H. Carpenter, of Oregon, and Dr. F. Pratt, of Michigan, member of the Judicial Council.

At 12 o'clock M., Vice-President Holton introduced Dr. John T. Hodgen, of St. Louis, the President of the Association, who proceeded to deliver the annual address.

### THE PRESIDENT'S ADDRESS.

*Colleagues of the American Medical Association:*

In obedience to the time-honored usage of the Association, I shall ask your attention to a few thoughts, such as may be supposed to befit the occasion of our coming together for our annual meeting. But, first, in the name of the Association, let me express to the local committee of arrangements, and to the medical professors and the citizens of Richmond, our grateful acknowledgments of the hearty reception which has been tendered by your Governor in this, the capital city of the oldest of American States, the historic mother of American Presidents.

The recent progress of medical science has been marked by exceptional strides, both in the direction of extending the domain and perfecting the methods of operative surgery. He alluded to the progress in medicine, and then proceeded to bestow a passing glance upon some of the causes which militate against the success of the surgeon by sometimes betraying him into error, again embarrassing him in his choice between conflicting plans of treatment, and too often frustrating his best directed efforts. He divided surgeons into those seeking to perform every practicable operation, and the other, avoiding operations whenever it is possible. The former include the bold, the enterprising, the ambitious and the reckless of our profession. The other, the timid, the conservative, the cautious and the procrastinating. The former class is largely made up of young men—enthusiastic and full of inspiration, caught from the professors whose task is to make the way clear and easy, students of the current medical literature, which teems with new suggestions and is crowded with reports of remarkable cases and wonderful operations, generally ending, or reported as ending, happily to the patient and to the great credit of the reporter.

Simon excises a kidney, turns an aberrant ureter into the rectum, touches, through the natural passages, a stone in the kidney; and immediately hundreds of ambitious surgeons are seeking kidneys to excise, ureters to turn, and renal calculi to touch. Battey removes an ovary for the relief of an obscure nervous disorder; and forthwith ovaries are removed for almost every imaginable nervous disorder. Billroth cuts out a cancerous larynx, or a diseased pylorus; and at once a demand springs up for similar cases, and the daring operations are repeated in all the four quarters of the globe.

The second class is recruited largely from the first, and often only after many lessons of bitter disappointment drawn from the experience of many and grave disasters.

The practice of seeking cases for operation and of operating by blindly following the dicta of authority, without a full understanding of the condition to be relieved, is well illustrated by two surgical procedures which have been resorted to, with far too great frequency, as I believe, by gynæcologists during the past and present decades. Of one of these procedures, the division of the cervix uteri for flexures, an operation without proper foundation in pathology, which was generally useless and often dangerous, and which always entailed deformity, Emmet speaks in the following energetic language: "Since the practice of indiscriminate division of the cervix was first introduced by Professor Simpson, more malpractice has been perpetrated throughout the world in the name of this simple operation, than from any other procedure known to the profession."

So, too, great wrong has been done in seeking to follow the lead of Dr. Emmet in the performance of operations for the cure of lacerations of the cervix uteri. From the large number of operations reported by many practitioners, it may be fairly concluded that it has often been needlessly and unprofitably performed.

A simple knowledge, however accurate, of the parts involved, does not qualify one to make an intelligent prognosis, to decide upon the advisability of an operation or treat judiciously even such diseases as consist mainly in pathological changes in the part in question—to say nothing of the many cases in which subjective symptoms are referred to a particular part, when they are in fact but the local expression of some remote or possibly constitutional trouble.

Herein lies a danger which threatens the profession, through the adoption of exclusive specialties by those not well trained in general medicine. It cannot be denied, that the early and exclusive study of the affections of a part tends to narrow the intellectual grasp and powers of the man who yields to the influences incident to such partial training. In the best sense, a specialist is a physician and something more; in the worst, he is something else and something less than a physician.

The rapid progress made of late years in the precision and perfection of regional surgery, the brilliant triumphs secured and the almost unlimited possibilities attained, combined to tempt surgeons to reckless operative procedures. Captivated by the knowledge that almost every region of the body has been and therefore may be invaded without necessarily destroying life, we are in danger of overlooking the general influences which are ever present to modify and control the results of local injuries.

The local conditions calling for surgical operations are, besides, more easily studied by the young surgeons than the general conditions which may forbid them, and are more fully discussed in the text books and college lectures. To learn what to do and how to do it, is always more attractive to the student than to be told what not to do. And this is especially true if the thing not to be done is something which he believes he can do well.

On the other hand, we recognize certain diseases and conditions, in which, however defective our knowledge may be in some respects, we are at least certain that very early operation is indicated, both as involving a minimum of risk, and as offering the best, or perhaps only chance, of saving life or of averting great calamity. In this class, we include tumors, benign or quasi-malignant.

The propriety of the early removal of quasi-malignant tumors is nowhere better illustrated than in the case of sarcoma of the choroid—a disease which, by the aid of the ophthalmoscope, can now be positively and accurately diagnosticated at a very early stage of development. Left to itself for a few months, it will surely break through the outer coat of the eyeball, and soon develop into a fatal and hideous tumor of the orbit, complicated probably with sarcomatous deposits in distant parts. Removed at an early stage by enucleation of the eyeball, it may never return *in situ*, and life may be indefinitely prolonged.

In rodent cancer and in epithelioma, we now expect a cure by excision, provided it is done early enough; and even in mammary scirrhus, removal of the breast has exceptionally effected a permanent cure.

Sympathetic ophthalmia affords a striking instance, which may result either from not recognizing a danger in season, or from a want of promptness in dealing with it.

Scarcely any fact is better established than that a high condition of health is not the condition which best fits the patient to bear the forced confinement, the impaired digestion, the imperfect assimilation and the perverted excretion which follow any serious bodily injury or grave surgical operation. In such patients, we have learned to dread surgical fever, and active inflammatory complications, leading possibly to septicæmia, and ending, it may be, in death.

So, too, that standard of health marked by an unusual ability to bear continuous mental strain, taxing the digestive and assimilative organs to their utmost, is not that under which the effects of shock are best borne, whether it be the shock of a severe injury or of a capital operation.

On the other hand, a man whose life is not marked by excessive tissue change, whose digestive, assimilative and excretory organs are not unduly taxed, and whose nervous system is not attuned to conditions of intense mental strain, is likely to bear well the shock of injury and the nutritive changes incident to prolonged confinement. Again, the chronic sufferer, whose nutritive and excretory organs have become educated, so to speak, to make good the excessive waste incident to any continuous drain, is often much better fitted to endure a grave surgical operation than is the new recruit of the army of sufferers. Very often the surgeon is compelled to act in the presence of morbid conditions of the most complex character.

Thus in strumous manifestations in connection whith chronic, suppurative disease of the joints and bones, the profuse discharge makes the most exacting demands upon the nutritive functions, while the close confinement, pain and loss of sleep unite to destroy the appetite and impair digestion and assimilation. In such a condition (so clearly set forth in the case of hip disease by our distinguished ex-President, Dr. Sayre), we recognize in the cachexia the effect rather than the cause of the local trouble; and by resection or amputation of the offending limb, we may arrest the exhausting discharge and restore the disturbed balances between the processes of nutrition and waste.

The dangers in certain depraved conditions of the body from injudiciously delaying an operation are forcibly depicted by Robert Barnes. He says: "My experience leads me to conclude that, in cases of urgent disease, there is more frequent occasion to regret having delayed the operation too long than having had recourse to it too soon.

"When, through obstinate vomiting, for example, nutrition has long been arrested, the starved tissues craving for supplies and falling into disintegration, feed the blood with depraved and noxious materials; the system feeds upon itself and poisons itself; the poisoned blood irritates the nervous centers, and these centers, wrought to a state of extreme morbid irritability respond to the slightest peripheral uterine or emotional excitation. All nervous energy is thus diverted from its destination, and exhausted in morbid action. Irritative fever ensues; the pulse rises to 140 or more; no organ of the body is capable of discharging its functions; for the pabulum of life is cut off at its very source. At this point, labor, whether it occurs spontaneously, as it often does, or be induced artificially, comes too late. The tissues are altered, the powers are impaired beyond recovery, and death soon follows."

Shock may act profoundly upon the whole economy. Arrested digestion, perverted assimilation, suspended secretion, and limited excretion may occur to vitiate the nutritive fluids of the body. Elements which should go to feed the tissues and provide materials for secretions remain unappropriated; excrementitious substances accumulate, and the body becomes gradually saturated with effete matters.

Operations for the relief of patients with old and tight urethral strictures, complicated with disease of the kidneys, afford illustration of the serious consequences which may follow shock in an already diseased organism. Internal division or forced dilatation of such a stricture may be attended with a degree of shock sufficient to arrest for the time the heart's action; or it may so act upon the whole nervous system as to check secretion and excretion generally. The diseased kidneys may thus cease altogether to perform their functions, leading to speedy death from uræmic poisoning; or, in the case of less aggravated renal trouble, the blood, becoming surcharged with morbid material, may no longer suffice to maintain the nervous centers in effective action ; assimilation, secretion and excretion may all fail, and death ensue from septicæmia.

Anæmia, resulting from sudden loss of blood, is particularly unfavorable to surgical interference ; besides the actual deficiency of blood, the diminished tension of the blood vessels favors the absorption of septic products at the site of the injury, while the blood, diluted and vitiated by the additional fluids absorbed from the tissues, becomes loaded with effete organic matter, ready to take on putrefactive changes. A familiar instance of susceptibility to septic influences after a large hæmorrhage will occur to every obstetrician who has learned how often metritis and septicæmia follow excessive *post-partum* hæmorrhage.

Besides want of space, another reason for the omission of reference to other conditions which may demand or forbid a resort to the knife, is our want of exact knowledge. Especially is this true of those constitutional conditions we term diatheses. Using the word in its broadest sense, diathesis is any condition varying from the normal standard which disposes to the development of disease in the presence of trivial exciting causes. Other condiditions which we habitually include under diathesis are themselves diseases—such, for instance, are scurvy, the scrofulous habit, tuberculosis and syphilis. A diathesis may be transient or permanent, retrogressive or progressive ; it may be so marked in its manifestations as to force its recognition upon even the most careless observer, or it may be so obscure as to elude the most painstaking scrutiny ; and yet it may respond immediately and disastrously to an injury. In acknowledging our ignorance regarding the precise nature of such variations from the normal standard as we believe must exist in diseases like scurvy, scrofula, tuberculosis, etc., we recognize the existence of wide, uncultivated fields, rich, no doubt, in promise to future investigators. A more perfect animal chemistry, a more thorough histology, and a deeper research into the possibilities of pathological change, will doubtless throw many a ray of light into regions where darkness is now too dense for our vision to penetrate. To these fields coming generations of physicians will surely be attracted, in the faith that as man advances in knowledge, and approaches somewhat nearer to the comprehension of the perfect wisdom which designed the wonderful physical organism, through which he is brought into relation with the world around him, he will be enabled to solve more and more of the difficult problems which now perplex and baffle us, and will gradually raise medicine to a position more nearly akin to that now accorded to the exacter sciences.

On motion of Dr. Brodie, the thanks of the Association were tendered the President for his address, and a copy of the same requested for publication.

### REPORT OF THE FOREIGN DELEGATES.

Dr. Joseph H. Warren, of Boston, Chairman of Committee of Foreign Delegates Abroad, presented his report. The reading of the report was deferred, and it was referred to the Publication Committee.

Below is an abstract of this report:

One of Dr. Warren's most important and pleasing duties was his attendance upon the 48th annual meeting of the British Association held on August 10, 11, 12, and 13th, in Cambridge. The meeting was very fully attended by the most noted and renowned men in the English profession, and some from other countries, and was said to be one of the largest and best gatherings of the kind held since the organization of the Association. Dr. Warren presented an abstract of the proceedings of the meeting and a short résumé of the different addresses. Among the more prominent were the address on Surgery, by Timothy Holmes, on "Fergusson and Conservative Surgery—Excision of the Knee and of the Hip;" the address in Physiology, by Michael Foster, on "Relations of Physiology and Pathology—The Professional Aspects of Physiology," and the address of Sir James Paget on "Elementary Pathology." The various sections were fully attended. Dr. Warren spoke of the address of Sir Henry Thompson, entitled "Remarks and Progress in Stricture of the Urethra," and also an address by the same surgeon upon the "Removal of Stone by Lithotrity." Dr. Warren himself showed some twelve or fifteen of his newly devised surgical instruments, and read a paper on "Operations for Cure of Hernia by Subcutaneous Injections," which seemed to be well received. In the Section of Physiology, there was an interesting discussion by Preyer, Brown-Séquard, Prof. Bowditch, of Boston, Dr. Beard, of New York, and others, upon that most recent study, "Sleep and Hypnotism." In Obstetric Medicine, the address by Playfair on "The Teaching of Obstetric Medicine" was very opportune, as showing that the instruction in this department should be much more thorough than they are wont to be. In the Section of the Diseases of Women, Dr. M. A. Pallen, of New York, read a paper on the "Etiology and Treatment of the Laceration of Cervix Uteri," a subject in which the English seem to display a want of knowledge.

After announcements by the Secretary of meetings to be held in the afternoon, the meeting adjourned till 10 o'clock Wednesday.

